# Prdx6 Regulates Nlrp3 Inflammasome Activation-Driven Inflammatory Response in Lens Epithelial Cells

**DOI:** 10.3390/ijms242216276

**Published:** 2023-11-13

**Authors:** Bhavana Chhunchha, Rakesh Kumar, Eri Kubo, Priyanka Thakur, Dhirendra P. Singh

**Affiliations:** 1 Department of Ophthalmology and Visual Sciences, University of Nebraska Medical Center, Omaha, NE 68198, USA; kumarrk26590@gmail.com (R.K.); pthakur@unmc.edu (P.T.); 2 Department of Ophthalmology, Kanazawa Medical University, Kahoku 9200293, Ishikawa, Japan; kuboe@kanazawa-med.ac.jp

**Keywords:** oxidative stress, Peroxiredoxin 6, NF-ĸB, Nlrp3, GSDMD, Caspase-1, IL-1β

## Abstract

The continuum of antioxidant response dysregulation in aging/oxidative stress-driven Nlrp3 inflammasome activation-mediated inflammatory response is associated with age-related diseases. Peroxiredoxin (Prdx) 6 is a key antioxidant that provides cytoprotection by regulating redox homeostasis. Herein, using lens epithelial cells (LECs) derived from the targeted inactivation of Prdx6 gene and aging lenses, we present molecular evidence that *Prdx6*-deficiency causes oxidative-driven Nlrp3 inflammasome activation, resulting in pyroptosis in aging/redox active cells wherein Prdx6 availability offsets the inflammatory process. We observed that *Prdx6*^−/−^ and aging LECs harboring accumulated reactive oxygen species (ROS) showed augmented activation of Nlrp3 and bioactive inflammatory components, like Caspase-1, IL-1β, ASC and Gasdermin-D. Similar to lipopolysaccharide treatment, oxidative exposure led to further ROS amplification with increased activation of the Nlrp3 inflammasome pathway. Mechanistically, we found that oxidative stress enhanced Kruppel-like factor 9 (Klf9) expression in aging/*Prdx6*^−/−^ mLECs, leading to a Klf9-dependent increase in Nlrp3 transcription, while the elimination of ROS by the delivery of Prdx6 or by silencing Klf9 prevented the inflammatory response. Altogether, our data identify the biological significance of Prdx6 as an intrinsic checkpoint for regulating the cellular health of aging or redox active LECs and provide opportunities to develop antioxidant-based therapeutic(s) to prevent oxidative/aging-related diseases linked to aberrant Nlrp3 inflammasome activation.

## 1. Introduction

Recent accumulating studies in the field of aging and age-related diseases authentically demonstrate that the etiopathogenesis of age-related diseases (ARD) is linked to the induction of inflammatory responses with advancing age [1,2,3,4,5]. Also, there is rising recognition that inflammasome activation by external or internal stimuli has similar pathogenic signaling, which leads to pathologies of a variety of human degenerative disorders compassing cardiovascular pathology, neurodegenerative and blinding diseases and aging itself [6,7,8,9,10,11]. The inflammasomes are cytoplasmic multiprotein complex sensors that can sense various injurious signals and in turn evoke inflammatory pathways by activating caspases (Casp), like Casp-1, and regulating proinflammatory cytokines interleukin (IL)-1β, IL-8 and IL-18 [12,13,14]. The Nlrp3 [nucleotide oligomerization domain (NOD)-like receptor family pyrin domain containing 3] inflammasome is widely studied and a crucial member of the NLR (NOD-like receptor) family, which plays a key role in the initiation of inflammatory signaling associated with aging or age-related diseases [15]. Similar to other inflammasomes, the Nlrp3 inflammasome acts as cytoplasmic sensor and is activated by a wide variety of stimuli. This includes extracellular ATP, toxins, ionophores, nigericin, a crystalline structure, and fatty acids as well as reactive oxygen species (ROS) [16,17,18,19,20]. These factors induce the recruitment of adaptor molecule ASC (apoptosis-associated speck-like protein) to produce inflammasome and caspase activation, like Casp-1 [21,22], resulting in Casp-mediated activation of proinflammatory cytokines like bioactive IL-1β, as well as cleavage of Gasdermin D (GSDMD), an executor of pyroptosis [23,24,25,26,27]. 

Inflammasome activation is a complex process, which includes changes in the levels of transcription of participating inflammatory components. There are several known stimuli, including ROS and Nlrp3, that enhance the transcription of the genes involved in the inflammasome pathway via activating transcriptional proteins [15,28,29]. In this context, redox-sensitive inflammatory signaling can activate the inflammasome via the ROS-driven NF-κB activation of proinflammatory cytokines, including Nlrp3 [30,31,32,33]. We have also shown that ROS-driven oxidative stress is a major molecular event in the activation of NF-κB-mediated aberrant redox signaling in *Prdx6*-deficient cells, and these cells go to spontaneous cell death and are prone to stressor-induced cell death [34,35,36]. The Nlrp3 inflammasome has been recognized to be the most vital target for delaying/treating several disorders, as its activation is evoked through a wide range of stress inducers, such as pathogens, toxins, degenerative/dead cells and damaged DNA/RNA aggregates, which are called pathogen-associated molecular patterns (PAMPs) or damage-associated molecular patterns (DAMPs) [17,22]. It has also been shown that multiple pathways and mechanisms can be involved in the activation of the Nlrp3 inflammasome depending upon cell/tissue types [37,38,39,40]. ROS have been identified to be one of the critical elements for Nlrp3 inflammasome activation [38,41]. It has been reported that dysregulation of antioxidant genes and ROS amplification occur due to suppression of the antioxidant gene by transcription factor Klf9 (Kruppel-like factor) [42,43,44,45], resulting in a sustained increase in ROS production and activation of the Nlrp3 inflammasome pathway. The transcription factor Klf9 (Kruppel-like factor 9) transmodulates genes with a single GC box or repeated GC boxes present in the proximal promoter region of genes [46,47]. Studies have shown the involvement of Klf9 in the regulation of a variety of biological activities, such as cell proliferation, DNA damage, apoptosis and the differentiation of various cell types [45,48,49]. Klf9 performs these functions by repressing or activating gene transcription. For example, Klf9 acts as a repressor by binding to a single GC box present in the gene promoter region, including antioxidant genes like Prdx6 [42,43,45,48,50]. Moreover, the aberrant expression of Klf9 has been reported during excessive oxidative stress, resulting in the suppression of antioxidant genes and the increased amplification of ROS and cell death [42,43,45]. Further, Klf9 knockdown reduces the expression of GSDMD in LPS-treated mice [51]. In addition, it has been found that LECs deficient in Klf9 confer resistance against oxidative stress [42,43].

To cope with oxidative damage and to protect the highly redox active cellular organelles, cells have evolved antioxidant defense systems, which includes a variety of antioxidant enzymes having specific protective functions with different and specific cellular localization patterns, such as glutathione peroxidases, catalases, peroxiredoxins, and superoxide dismutase [52,53,54,55,56]. Increased ROS production from organelles due to reduced levels of antioxidants results in oxidative-evoked danger signaling, leading to inflammasome activation [30,33,57]. There are several cellular activators of Nlrp3, such as lysosomal damage and the excessive production of ROS from mitochondria, the endoplasmic reticulum, and the plasma membrane [35,36,38,41,58,59,60,61]. Recently, several studies have shown that the delivery of antioxidants like NAC (N-acetyl cysteine) can impair the Nlrp3 inflammasome activation pathway [62,63,64]. Prdx6 has multiple protective functions and maintains cellular integrity and redox homeostasis by its GSH peroxidase [4,5,65], acidic Ca^2+^-independent phosphatase A_2_ (iPLA_2_) [53,54] and lysophosphatidylcholine acyltransferase (LPCAT) [56] activities. Thus, Prdx6 virtually has a unique ability to regulate cellular signaling, including the maintenance of phospholipid turnover and quality control. Furthermore, its reduced expression results in cell death, inflammatory death signaling, tissue degeneration and the progression of several diseases, including cataractogenesis [4,5,66,67,68,69]. However, ROS are maximally produced by mitochondria, the endoplasmic reticulum, the plasma membrane, lysosomes so on [52,70,71,72,73]. Importantly, Prdx6 is localized in these compartments to optimize ROS in favor of cell health. Thus, the loss of Prdx6 in these organelles, specifically in mitochondria and lysosomes, can lead to their disintegration and dysfunction, resulting in the excessive ROS-mediated release of molecules related to the generation of inflammatory responses [71,74,75]. Recent studies have shown that the destabilization of lysosome and mitochondrial machinery is a cause for ROS-DAMPs/PAMPs-mediated Nlrp3 inflammasome activation [57,76,77,78,79,80,81].

In this study, using lens epithelial cells of variable ages coupled with redox active *Prdx6*-deficient LECs, we showed that *Prdx6*-deficiency in LECs led to Nlrp3 inflammasome activation-mediated signaling, resulting in the inflammatory form of cell death, pyroptosis. We found that the unphysiological regulation of Nlrp3 inflammasome activation-mediated inflammatory signaling was connected to the Klf9-dependent excessive accumulation of intracellular ROS levels. The process was further aggravated by Nlrp3 inflammasome inductors, like LPS or H_2_O_2_ [82,83], while the inflammatory process could be impeded by delivering the antioxidant Prdx6 to cells, and argue that Prdx6 is pivotal to attenuate the Nlrp3 inflammasome activation pathway. Mechanistically, we identified that the aberrant expression of Klf9 in redox active or aging LECs results in the direct repression of antioxidant gene transcription, leading to ROS amplification. We observed the existence of the Klf9-dependent feedforward production of ROS within a cellular microenvironment, leading to a Klf9-mediated increased expression of the Nlrp3 inflammasome-mediated amplification of the inflammatory form of cell death. Altogether, this study for the first time shows that Nlrp3 is a target gene for Klf9. Also, our findings suggest that the delivery of Prdx6 or Klf9/Nlrp3 inhibitors should be a potential therapeutic strategy for the prevention/treatment of the inflammasome activation-mediated inflammatory signaling involved in the etiopathogenesis of age-related diseases.

## 2. Results

### 2.1. Prdx6-Deficient mLECs Having Increased ROS Accumulation Displayed Aberrant Expression and Activation of Nlrp3 Inflammasome, Bioactive Caspase-1, IL-1β, IL-18 and GSDMD

**Figure 1:**
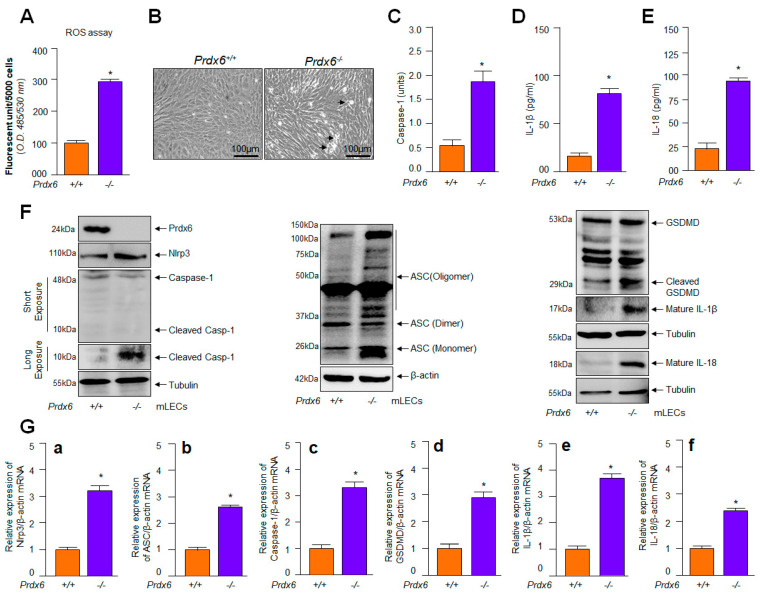
Increased oxidative load in redox active *Prdx6*^−/−^ mLECs was connected to increased expression of Nlrp3, cleaved Casp-1, and mature form of IL-1β and IL-18. (**A**) *Prdx6*−deficient mLECs showed increased expression of ROS. ROS levels were quantified using H_2_-DCF-DA dye. Data represent the mean ± S.D. of three independent experiments. *Prdx6*^+/+^ vs. *Prdx6*^−/−^ mLECs samples; * *p* < 0.001. (**B**) Photomicrographs of *Prdx6*^+/+^ and *Prdx6*^−/−^ mLECs. *Prdx6*^−/−^ mLECs displayed spontaneous cell death, shown with arrow heads. (**C**) *Prdx6*^−/−^ LECs displayed elevated Casp-1 activity. *Prdx6*^+/+^ and *Prdx6*^−/−^ mLECs were cultured and 48 h later cellular extracts were prepared. The extracts were used to examine Casp-1 activity. Data represent the mean ± S.D. from three independent experiments. *Prdx6*^+/+^ vs. *Prdx6*^−/−^ mLECs samples; * *p* < 0.001. (**D**,**E**) *Prdx6*^−/−^ LECs showing increased secretion of mature IL-1β and IL-18 in cell culture supernatant. mLECs were cultured; 48 h later supernatant was collected and we measured the levels of IL-1β (**D**) and IL-18 (**E**). Data represent the mean ± S.D. from three independent experiments. *Prdx6*^+/+^ vs. *Prdx6*^−/−^ mLECs samples; * *p* < 0.001. (**F**,**G**) Increased expression of Nlrp3 inflammasome, ACS, Casp-1, GSDMD, inflammatory cytokines, IL-1β and IL-18 in *Prdx6*^−/−^ mLECs. (**F**) Total protein was isolated from *Prdx6*^+/+^ and *Prdx6*^−/−^ mLECs and subjected for Western blot, as indicated in figure. Tubulin and/or β–actin served as a loading control. (**G**) Total RNA was isolated from *Prdx6*^+/+^ and *Prdx6*^−/−^ mLECs and submitted for qPCR, as indicated in figure. β-actin was used as a control. (**a**), Nlrp3 mRNA; (**b**), ASC mRNA; (**c**), Caspase-1 mRNA; (**d**), GSDMD mRNA; (**e**), IL-1β mRNA and (**f**), IL-18 mRNA. Data represent the mean ± S.D. from three independent experiments. *Prdx6*^+/+^ vs. *Prdx6*^−/−^ mLECs, * *p* < 0.001.

The cellular expression of multitasking biomolecule Prdx6 is vitally important for the maintenance of redox homeostasis, quality control of cellular organelles and cellular protection against intrinsic or extrinsic stressors [35,52,75,76]. Conversely, a loss of Prdx6 leads to increased ROS-driven cellular abnormality and spontaneous cell injuries [34,35,36]. To determine whether Prdx6 plays a crucial role in the regulation of the Nlrp3 inflammasome activation-mediated inflammatory pathway, we utilized *Prdx6*-deficient (*Prdx6*^−/−^) LECs and examined the biological status of components of Nlrp3 inflammasomes and determined their biological patterns in connection to ROS and Prdx6 prevalence. Toward this, we isolated LECs from *Prdx6* knockout (*Prdx6*^−/−^) and wild-type (*Prdx6*^+/+^) mice eye lenses, as described in ‘Materials and Methods’. Because ROS are suggested to be involved in the regulation of the Nlrp3 inflammasome activation pathway, we quantified ROS using H_2_-DCF-DA dye. As expected, we observed that *Prdx6*^−/−^ mLECs could bear significantly higher levels of ROS (Figure 1A) and our careful microscopic observation revealed that these cells went to spontaneous death (Figure 1B). Furthermore, as noted in the Introduction section, excessive ROS levels, and ROS-driven DAMP and PAMP improperly regulate Nlrp3 inflammasome activation, and this includes bioactive Casp-1 and proinflammatory cytokine release and GSDMD. Next, we tested the secretion levels of IL-1β and IL-18 and found that both molecules were significantly increased in cultured supernatants of *Prdx6*-deficient LECs compared to wild-type LECs (Figure 1D,E). Further, this is because Casp-1 (Figure 1C) in the Nlrp3 inflammasome complex is responsible for processing the proinflammatory cytokines to bioactive cytokines, and cleaves GSDMD to form pores in the plasma membrane and induce a proinflammatory form of cell death, pyroptosis [27,84]. Next, in parallel set of experiments, we examined whether *Prdx6*-deficient mLECs produce a higher amount of bioactive fragments of inflammatory components and ASC expression with a dimer/trimer. Immunostaining of these molecules with their corresponding antibodies, as indicated in Figure 1F, revealed that the protein levels of these inflammatory components were significantly increased, with cleaved bioactive fragments and increased oligomeric forms of ASC in *Prdx6*^−/−^ compared to *Prdx6*^+/+^ LECs. Furthermore, considering that the expression of the Nlrp3 inflammasome complex increased in *Prdx6*-deficient LECs, next we wished to know whether the increased expression observed at the protein levels could be related to their enhanced transcription. As expected, qPCR analysis revealed a significant increase in the expression of Nlrp3 and its components’ transcripts (Figure 1G(a–f)) in *Prdx6*-deficient LECs, suggesting that the induction of transactivation machinery played a role in the increase in the transcription of the Nlrp3 inflammasome and its related inflammatory components, as reported for other cell types [28,62,85]. These results indicate that Prdx6 expression is critical to optimize the Nlrp3 inflammasome activation-mediated inflammatory death pathway, while its deficiency is the cause of the activation of inflammatory responses, at least in LECs, suggesting that Prdx6 is required to inhibit the aberrant transcriptional induction and production of bioactive inflammatory components related to Nlrp3 inflammasome-mediated inflammatory responses.

### 2.2. Age-Dependent Increased Levels of Bioactive Nlrp3, ASC, Cleaved Casp-1 and Inflammatory Cytokines Were Related to Progressive Loss of Prdx6 with Increased Accumulation of ROS

**Figure 2:**
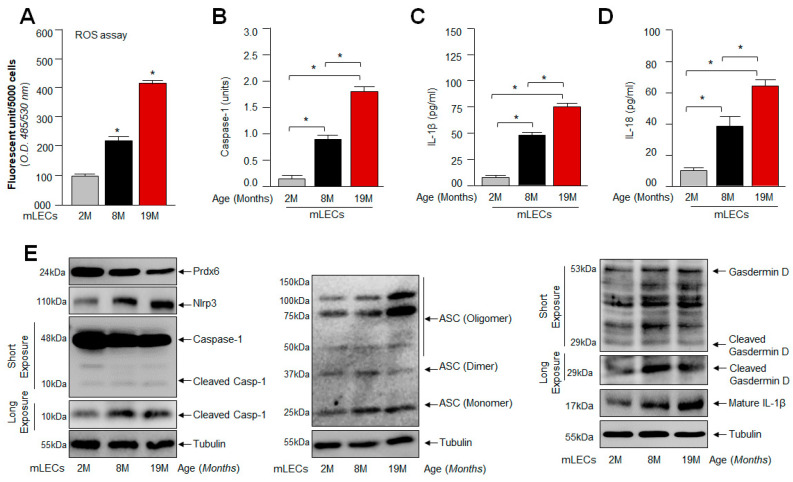
Increased ROS level associated with the aberrant expression of Nlrp3 inflammasome-associated genes with advancing age of mouse LECs. (**A**) ROS levels were measured in intact lenses isolated from different ages of mice using H2-DCF-DA dye method. Data represent the mean ± S.D. from three independent experiments. mLEC samples at 2 M vs. 8 M and 19 M; 8 M vs. 19 M; * *p* < 0.001. (**B**) Aging LECs displayed progressive increase in Casp-1. A cellular extract was prepared, and the Casp-1 level was assayed. Data represent the mean ± S.D. from three independent experiments. mLEC samples at 2 M vs. 8 M and 19 M; 8 M vs. 19 M; * *p* < 0.001. (**C**,**D**) Increased secretion of mature IL-1β and IL-18 observed in aging LECs. mLECs were cultured, and after 48 h collected supernatant was measured for IL-1β (**C**) and IL-18 (**D**) levels. Data represent the mean ± S.D. from three independent experiments. mLEC samples at 2 M vs. 8 M and 19 M; 8 M vs. 19 M; * *p* < 0.001. (**E**) Aging mLECs showed increased expression of Nlrp3, ASC, cleaved Casp-1 and mature IL-1β. (**E**) Total protein was isolated from aging mLECs and subjected to Western blot as indicated. Tubulin served as internal control.

Recent studies have demonstrated that aberrant activation of Nlrp3 inflammasome-mediated inflammatory injurious signaling in response to the excessive accumulation of ROS, due to a deteriorating antioxidant defense system in aging, is related to the development of aging-associated diseases [15,59,86]. Previously, we also reported that the age-dependent loss of Prdx6 is connected to the excessive accumulation of ROS and failure of redox homeostasis in mLECs [4,5,35,43]. However, based on the results in Figure 1 showing that the increased ROS accumulation in *Prdx6*^−/−^ mLECs, a model for aging [4,5], was directly linked to the expression and activation of the Nlrp3 inflammasome-mediated inflammatory response in these cells, compared to mLECs expressing Prdx6, we asked the question whether a progressive increase in ROS in aging LECs due to *Prdx6*-deficiency leads to Nlrp3 inflammasome activation and bioactive inflammatory molecules. To address this, we isolated mLECs from different ages of C57BL/6 mice and measured their intracellular levels of ROS. Quantitation by staining with H_2_-DCF-DA established a higher prevalence of ROS with advancing age (Figure 2A). However, it is notable that DCF florescence is not specific for H_2_O_2_, and other ROS such as peroxinitrite, O_2_^−^, NO, etc., can also oxidize DCFH_2_ into DCF. Hence, DCF fluorescence reflects the overall oxidative load in cells [87]. We observed an increased intracellular ROS level with advancing age (Figure 2A). Next, we wished to examine if aging mLECs activated the Nlrp3 inflammasome pathway, as reported in other cell types [74,88,89]. So, to investigate specifically the connection between increased ROS levels with aging (Figure 2A) and the Nlrp3 inflammasome activation pathway in these cells, in parallel set of experiments, we employed a biochemical test, protein blot and ELISA analyses. Figure 2 showed a progressive increase in the expression of Nlrp3 with activated Casp-1, the enhanced secretion of IL-1β and IL-18 and the expression of ASC (monomer, dimer, and oligomer) in mLECs with aging (Figure 2B–E). As expected, we found that the increased Nlrp3 inflammasome activation inflammatory pathway was directly related to the increase in ROS accumulation in mLECs (Figure 2A). In addition, our data disclosed that the progressive increase in ROS in aging mLECs is linked to the deterioration of Prdx6, as indicated in Figure 2E, Panel Prdx6. Taken together, the data showed that aging mLECs (directly separated from lens to avoid the cell-culture effect) suffered from the Nlrp3 inflammasome activation-driven inflammatory response. Backed by these findings, we posit that Nlrp3 inflammasome activation-mediated adverse signaling may be a critical event in the onset of aging pathology in the eye lens, as reported in other model systems.

### 2.3. Similar to mLECs, Aged hLECs Had Increased Expression and Activation of Nlrp3 Inflammasome Components, Including Inflammatory Bioactive Molecules, Casp-1 and IL-1β and IL-18 and GSDMD

**Figure 3:**
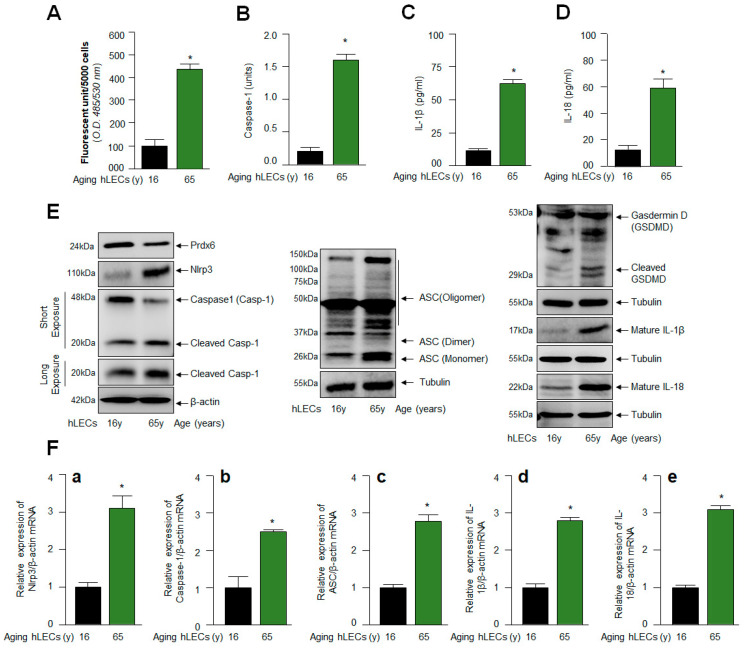
Age-related increase in Casp-1 activation, and cytokines IL-1β and IL-18 were regulated via the activation of the Nlrp3 inflammasome. (**A**) H_2_-DCF-DA dye was used to measure the ROS levels in human aging lens/LECs, as indicated in the figure. Data represent the mean ± S.D. from three independent experiments. hLECs samples aged 16 y (n = 3) vs. 65 y (n = 3); * *p* < 0.001. (**B**) Aging hLECs displayed elevated Casp-1 activity. The cellular extract was prepared from different ages of hLECs and used to examine Casp-1 activity. Data represent the mean ± S.D. from three independent experiments. hLECs samples aged 16 y vs. 65 y; * *p* < 0.001. (**C**,**D**) Aging hLECs showed increased levels of mature IL-1β and IL-18. Aging hLECs were cultured, and 48 h later supernatant was collected and measured the level of IL-1β (**C**) and IL-18 (**D**). Data represent the mean ± S.D. from three independent experiments. Young vs. aging hLECs samples, * *p* < 0.001. (**E**) Aging LECs showed increased expression of Nlrp3, cleaved Casp-1 and IL-1β and IL-18. Total cell lysate was prepared from aging hLECs and subjected to Western blot analysis to assess the levels of Nlrp3, ASC, Casp-1, pyroptosis inducer GSDMD, and IL-1β using corresponding antibodies as indicated. Tubulin/β-actin was used as loading control. (**F**) Total RNA was isolated from young and aged hLECs and subjected for mRNA level using qPCR, as indicated in figure. β-actin was used for normalization. (**a**), Nlrp3 mRNA; (**b**), Caspase-1 mRNA; (**c**), ASC mRNA; (**d**), IL-1β mRNA and (**e**), IL-18 mRNA. Data represent the mean ± S.D. from three independent experiments. 16 y vs. 65 y, * *p* < 0.001.

Several recent reports have shown that ROS drive Nlrp3 inflammasome activation-mediated-aging-related pathologies and diseases [16,33,90,91,92]. However, recent studies have shown that Nlrp3 inflammasome activation can be species- and cell-type-specific [41]; hence, we wanted to know whether aged hLECs derived from the lenses of deceased healthy human subjects have a higher prevalence of the aberrantly activated Nlrp3 inflammasome and its bioactive components, with reduced levels of Prdx6 and accumulated intracellular ROS, as observed in aging mLECs and *Prdx6*-deficient mLECs. As described above in Section 2.2, we examined the levels of Nlrp3 inflammasome activation, activation of Casp-1, IL-1β and IL-18 in aging LECs. After quantitation of ROS levels using H2-DCF-DA dye, our data revealed that aged hLECs had a higher accumulation of ROS compared to younger hLECs (Figure 3A). In a parallel set of experiments, a supernatant was collected from cultured aging hLECs, and then cellular extracts were prepared from those hLECs and processed for expression and ELISA assays (Figure 3B–E). Similar to aging mLECs, aged hLECs (green bar; 65 y) displayed increased expression and activation of the Nlrp3 inflammasome and the bioactive inflammatory components ASC, Casp-1, GSDMD and IL-1β, with increased secretion of IL-1β (Figure 3C) and IL-18 (Figure 3D), and this inflammatory phenomenon was directly related to a significant reduction in Prdx6 and increased accumulation of ROS (Figure 3A,E). In another set of experiments, qPCR with total RNA isolated from younger and aged cells (16 y and 65 y) showed that the increased expression of inflammatory molecules was at transcriptional levels (Figure 3F(a–e)), as reported earlier for other aging or redox active cell types [31,32,74]. Collectively, our results revealed that LECs derived from mouse or human subjects express increased levels of the Nlrp3 inflammasome and its components, as well as these being aberrantly activated during aging, leading to the inflammatory form of cell death. It is noteworthy that pyroptosis occurs upon activation of proinflammatory cytokines, bioactive Casp-1 and GSDMD [27,93]. Thus, our results indicated that *Prdx6*-deficiency in aging is a cause for inflammatory cell death, pyroptosis, and is related to the increased prevalence and accumulation of intracellular ROS due to a loss of Prdx6.

### 2.4. Prdx6-deficient mLECs and Prdx6 Expressing mLECs under Extrinsic Oxidative Stimulus Showed Aberrant Nlrp3 Inflammasome Activation-Mediated Signaling

**Figure 4:**
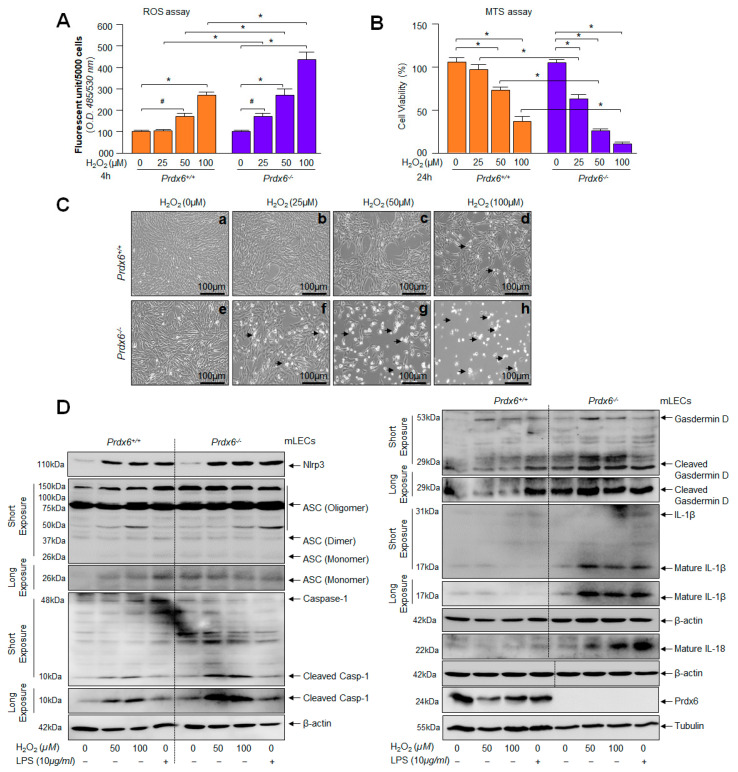
(**A**) Redox active *Prdx6*^−/−^ mLECs displayed increased sensitivity to H_2_O_2_-induced oxidative stress. H2-DCF-DA dye was added to H_2_O_2_-treated *Prdx6*^+/+^ and *Prdx6*^−/−^ mLECs, and fluorescent units were recorded. Significantly increased ROS levels were observed in *Prdx6*-deficient mLECs exposed to different concentrations of H_2_O_2_ in a dose-dependent fashion. Data represent the mean ± S.D. from three independent experiments. (**B**) Cell viability assay showing higher vulnerability of *Prdx6*^−/−^ cells to H_2_O_2_ exposure. *Prdx6*^+/+^ and *Prdx6*^−/−^ mLECs were treated with different concentrations of H_2_O_2_. MTS assay was performed after 24 h, as indicated. Compared to *Prdx6*^+/+^ cells, *Prdx6*^−/−^ cells were found to be more susceptible to H_2_O_2_-induced cell death. Results were normalized according to the absorbance of the untreated control. Data represent the mean ± S.D. from three independent experiments (# *p*< 0.05, * *p* < 0.001). (**C**) Photomicrograph of *Prdx6*^+/+^ and *Prdx6*^−/−^ mLECs with or without H_2_O_2_ exposure. Cells were treated with different concentrations of H_2_O_2_ (25, 50 and 100 µM) for 24 h. Significant cell death (rounded white cells, arrowhead) were observed in *Prdx6*^−/−^ cells exposed to H_2_O_2_ (bottom: e, 0 µM H_2_O_2_; f, 25 µM H_2_O_2_; g, 50 µM H_2_O_2_; h,100 µM H_2_O_2_) while no significant cell death occurred in *Prdx6*^+/+^ exposed with H_2_O_2_ (top: a, 0 µM H_2_O_2_; b, 25 µM H_2_O_2_; c, 50 µM H_2_O_2_; d, 100 µM H_2_O_2_). (**D**) mLECs facing oxidative stress showed the increased expression of Nlrp3 inflammasome-mediated signaling components, ASC and activated Casp-1, increased GSDMD, and the enhanced expression of cytokines such as IL-1β and IL-18. *Prdx6*-deficient mLECs showed higher susceptibility to H_2_O_2_ exposure and this led to Nlrp3 inflammasome-mediated signaling. *Prdx6*^+/+^ and *Prdx6*^−/−^ mLECs were exposed to different concentrations of H_2_O_2_ or LPS for 24 h in serum-free DMEM medium. Total lysates were prepared and subjected to Western blot analysis to evaluate the protein expression of Nlrp3, Casp-1, ASC, GSDMD, and mature form of IL-1β and IL-18, using their specific antibodies. β-actin was used as an internal control.

From the above results (Figure 1, Figure 2 and Figure 3), it is evident that *Prdx6*-deficiency is a cause for Nlrp3 inflammasome activation-mediated inflammatory responses. However, several reports have also shown that oxidative stress and aging diseases share similar proinflammatory signaling pathways [16,33,90,91,92], and oxidative stress-induced by extrinsic stimuli further amplify intrinsically activated the Nlrp3 inflammasome activation pathway, as noted in the Introduction section. Thus, we asked the question whether oxidative stress-generated by an external stimulus initiates the Nlrp3 inflammasome activation pathway in Prdx6-expressing mLECs and also accelerates further the intrinsically primed Nlrp3 inflammasome improper activation in *Prdx6*-deficient mLECs. To achieve this, first we determined the levels of ROS and cell viability exposed to H_2_O_2_-induced oxidative stress. As expected, we observed that *Prdx6*^−/−^ mLECs exposed to H_2_O_2_ displayed the higher accumulation of ROS with significantly reduced cell viability (Figure 4A,B). Photomicrographs showed increased cell death (rounded white dead cells) in *Prdx6*^−/−^ than *Prdx6*^+/+^ LECs. The results also showed that *Prdx6*^−/−^ mLECs could not resist H_2_O_2_ at even lower concentrations, while *Prdx6*^+/+^ mLECs engender resistance against H_2_O_2_-induced cytotoxicity (Figure 4C). In another set of parallel experiments, we assessed whether H_2_O_2_ or LPS (positive control vehicle) treatments could amplify Nlrp3 inflammasome signaling in *Prdx6*-deficient mLECs. We exposed the mLECs to variable concentrations of H_2_O_2,_ as well as a positive control, LPS (Figure 4D). The data disclosed that H_2_O_2_ treatment augmented the activation and expression of the Nlrp3 inflammasome and its inflammatory bioactive proteins similar to LPS. However, *Prdx6*^−/−^ cells were more sensitive to oxidative stress and had elevated Nlrp3 inflammasome activation inflammatory signaling and increased cell death, as observed in Figure 4C(e–h), compared to Prdx6 expressing mLECs. Taken together, our results indicate that the accumulation of ROS in *Prdx6*-deficient cells and Prdx6 suppression in H_2_O_2_-treated mLECs expressing Prdx6 are major events for the activation of the Nlrp3 inflammasome-mediated death pathway, and demonstrate that Prdx6 is an essential molecule to regulate this inflammatory injurious signaling.

### 2.5. SRA-hLECs under Oxidative Stress Showed Aberrant Nlrp3 Inflammasome Activation and Its Components, Bioactive ASC, Activated Casp-1 and GSDMD, and Enhanced Release of IL-1β and IL-18

**Figure 5:**
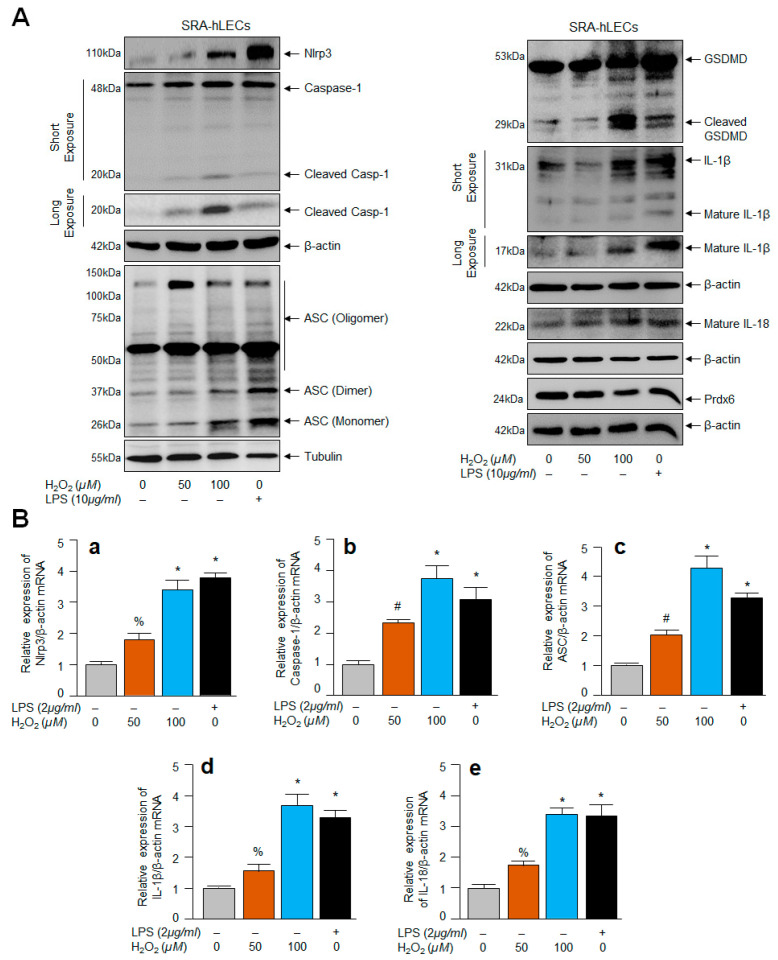
SRA-hLECs facing oxidative stress displayed the increased expression of Nlrp3, Casp-1, ASC, GSDMD, and cytokines, IL-1β and IL-18. SRA-hLECs were exposed to different concentrations of H_2_O_2_ or LPS for 24 h in serum free DMEM medium. Total protein and RNA were isolated from SRA-hLECs facing oxidative stress and subjected to Western blot (**A**) and mRNA (**B**) to assess the expression and status of Nlrp3, Casp-1, ASC, pyroptosis executer, GSDMD, and cytokines such as IL-1β and IL-18 using their specific probes as indicated. β-actin/Tubulin was used as an internal loading control. (**a**), Nlrp3 mRNA; (**b**), Caspase-1 mRNA; (**c**), ASC mRNA; (**d**), IL-1β mRNA and (**e**), IL-18 mRNA. Data represent the mean ± S.D. from three independent experiments. Control vs. H_2_O_2_ or LPS; % *p* < 0.01, # *p* < 0.05, * *p* < 0.001.

Several studies were conducted using the cell line of hLECs (SRA-hLECs) and they were been found to be responsive as primary hLECs, and SRA-hLECs show similar characteristics in response to oxidant treatment and provide similar outcomes for other biochemical experimentation as those observed in primary hLECs [4,5,65]; therefore, we used these cells to conduct some of the experiments in this study, as aging hLECs are scarce, and therefore precious. However, it is not known to us whether these SRA-hLECs, when treated with an oxidant like H_2_O_2,_ display an Nlrp3 inflammasome activation-mediated inflammatory response. To examine this, SRA-hLECs were exposed to different concentrations of H_2_O_2_ or LPS (as a positive control). The protein extracted and RNA isolated were processed for Western blot (Figure 5A) and qPCR (Figure 5B). The data revealed that SRA-hLECs are responsive and displayed H_2_O_2_ concentration-dependent aberrant expression and activation of the Nlrp3 inflammasome and its inflammatory bioactive components Casp-1, ASC, GSDMD, IL-1β or IL-18, as evidenced by Western blot and qPCR experiments (Figure 5A,B). These results indicated that SRA-hLECs could be utilized for the experimentation in the current study, as results obtained from these cells were similar to primary aging hLECs or mouse LECs. Nevertheless, to explore how intracellular ROS could be amplified in aging LECs or LECs under oxidative stress and what the underlying mechanism is, based upon our work and the work of other investigators showing that Klf9 acts as a suppressor of antioxidant genes [42,43,45,48], we examined the role of the transcription factor Klf9, if any, and carried out proceeding experiments, as noted below.

### 2.6. Enhanced Klf9 Levels in Aging LECs and Prdx6^−/−^ mLECs Were Related to Antioxidant Gene Repression

**Figure 6:**
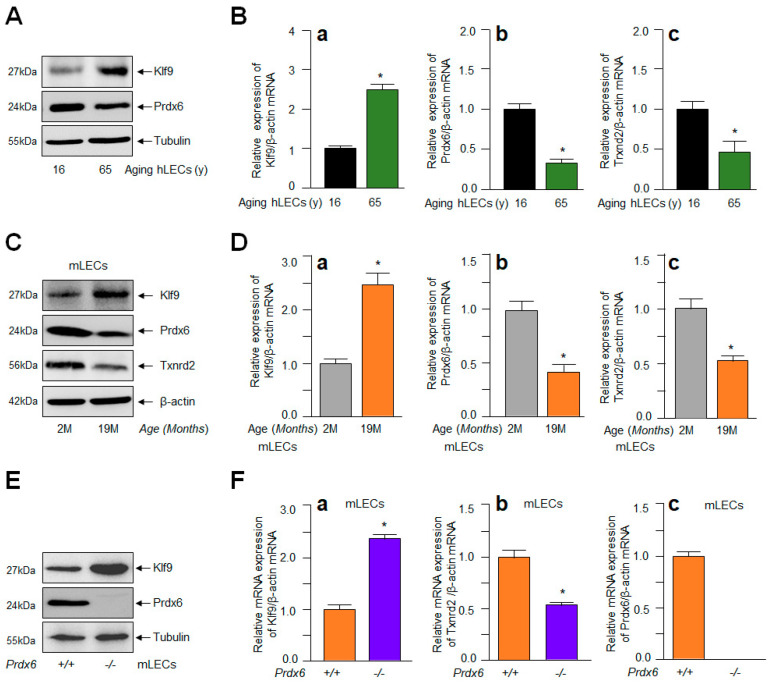
Aberrant expression of Klf9 in aging LECs and *Prdx6*^−/−^ was related to antioxidant gene repression. (**A**,**B**) Total protein and RNA were isolated from variable ages of hLECs and subjected for protein and mRNA expression using Western blot and qPCR with specific probes, respectively, as indicated. (**a**), Klf9 mRNA; (**b**), Prdx6 mRNA; (**c**), Txnrd2 mRNA. Data represent the mean ± S.D. from three independent experiments. hLECs samples aged 16 y vs. 65 y; * *p* < 0.001. (**C**,**D**) Total protein and RNA were isolated from C57BL/6 mouse (m) [2 months (2 M) and 19 M] lenses/LECs and processed for Western blot (**C**) and RT-qPCR (**D**) analysis with specific probes, respectively. (**a**), Klf9 mRNA; (**b**), Prdx6 mRNA; (**c**), Txnrd2 mRNA. Data represent the mean ± S.D. from three independent experiments. mLEC samples at 2 M vs. 19 M; * *p* < 0.001. (**E**,**F**) Total protein and RNA were isolated from *Prdx6*^+/+^ and *Prdx6*^−/−^ mLECs and Western blot and RT-qPCR analyses with specific probes were conducted. (**a**), Klf9 mRNA; (**b**), Prdx6 mRNA; (**c**), Txnrd2 mRNA. Data represent the mean ± S.D. from three independent experiments. *Prdx6*^+/+^ and *Prdx6*^−/−^ mLECs samples; * *p* < 0.001.

Recent works from our lab and other investigators have shown the Klf9-dependent suppression of some of the major antioxidant genes such as Prdx6 and Txnrd2, resulting in ROS amplification in response to increased oxidative load [42,43,45]. To examine if aging LECs display the Klf9-dependent suppression of antioxidants, resulting in ROS amplification, we generated aging LECs from the eye lenses of healthy deceased human subjects and C57BL/6 mice. In parallel experiments, we also utilized *Prdx6*^−/−^ mLECs (redox-active cells). The integrity of LECs were validated through protein blot using αA-crystallin antibody, a specific marker for LECs before experimentation. We also examined the identity/integrity of aging LECs using Pax6 or fibronectin [34,35,36,94] for the normalization of data following published protocols [36,94,95]. To examine Klf9 levels and its link to Prdx6 and Txnrd2 protein and mRNA, cellular extract (Figure 6A,C,E) and total RNA (Figure 6B,D,F) were isolated from aging LECs, as well as *Prdx6*^−/−^ mLECs. As shown in Figure 6, protein blot and qPCR analyses revealed that the aberrant expression of Klf9 in hLECs or mLECs was directly connected to the suppression of Prdx6 and Txnrd2 (aging hLECs, Figure 6A,B; aging mLECs, Figure 6C,D; *Prdx6*^−/−^ mLECs, Figure 6E,F) The results of this experimentation indicated the plausible involvement of Klf9 in the repression of major antioxidants at transcription levels that, in turn, leads to the accelerated accumulation of ROS within the cellular microenvironment, and provided a base for further experimentation.

### 2.7. Klf9 Overexpression in LECs Caused Elevated Oxidative Load, and the Aberrant Expression of Nlrp3 with Reduced Expression of Antioxidant Genes

**Figure 7:**
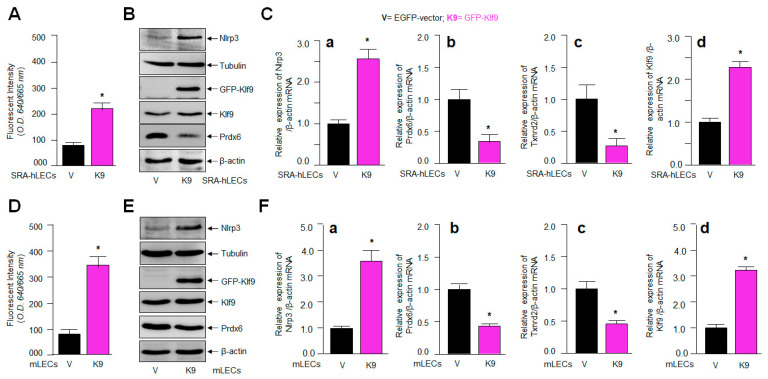
LECs overexpressing Klf9 showed increased oxidative load and Nlrp3 expression with the reduced expression of antioxidant genes. (**A**,**D**) LECs overexpressed with GFP-Klf9 displayed increased ROS levels. CellROX deep red dye was added to SRA-hLECs overexpressing Klf9 (**A**) and mLECs (**D**) and fluorescence intensity were recorded. Significantly increased ROS levels were detected, as indicated. Data represent the mean ± S.D. from three independent experiments. pGFP-vector vs. pGFP-Klf9 transfected LECs, * *p* < 0.001. (**B**–**F**) LECs overexpressing Klf9 had increased Nlrp3 expression with the reduced expression of the antioxidant genes examined, such as Prdx6 and Txnrd2. LECs were transfected with pGFP empty-vector or pGFP-Klf9. After 48 h, the total protein (**B**,**E**) and RNA (**C**,**F**) were isolated and processed for Western blot and qPCR analyses using specific probes, respectively. (**Ca**,**Fa**), Nlrp3 mRNA; (**Cb**,**Fb**), Prdx6 mRNA; (**Cc**,**Fc**), Txnrd2 mRNA; (**Cd**,**Fd**), Klf9 mRNA. Data represent the mean ± S.D. from three independent experiments. pGFP-vector (V) vs. pGFP-Klf9 (K9) transfected LECs, * *p* < 0.001.

The results from Figure 6 demonstrated that the increased Klf9 expression was linked to the suppression of Prdx6, resulting in ROS amplification in aging LECs and *Prdx6*-deficient mLECs; therefore, we intended to clarify whether the extrinsic overexpression of Klf9 in LECs is a cause for Prdx6 suppression with ROS amplification, resulting in aberrant Nlrp3 levels. Hence, at first, we assessed the intracellular ROS levels in SRA-hLECs (Figure 7A) as well as in mLECs (Figure 7D) overexpressed with Klf9. Quantitation of ROS in these transfectants showed a dramatic increase in ROS levels. Next, we measured the expression levels of Klf9 and the antioxidant gene Prdx6 in LECs overexpressing Klf9. Total protein (Figure 7B,E) and RNA (Figure 7C,F) were isolated from LECs overexpressing Klf9. Protein blot and qPCR data revealed that the expression of antioxidant genes such as Prdx6 and Txnrd2 protein and mRNA was significantly decreased. In contrast, data showed that the expression of Nlrp3 protein and mRNA was dramatically increased in LECs overexpressing Klf9. Because an increase in Nlrp3 with an increase in Klf9 could be at mRNA in LECs (Figure 7C(a–d),F(a–d)), we posit that Klf9 could be a new regulator for Nlrp3 gene transcription. Next, to address this question, we performed transactivation experiments, to know whether Klf9 over- or under-expression modulate Nlrp3 gene transcription, in Section 2.8 below.

### 2.8. Loss and Gain Experimentation with Klf9 Disclosed That the Nlrp3 Gene Could Be a New Target for Klf9-Mediated Transcription

**Figure 8:**
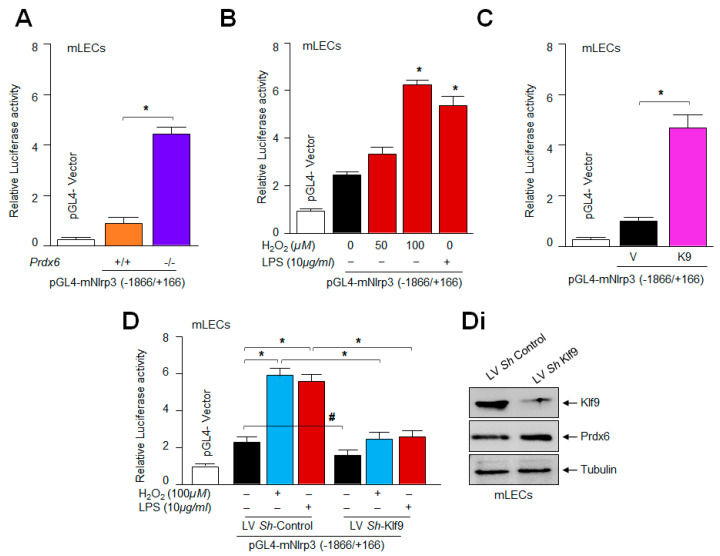
Prevalence of Klf9 in LECs determined Nlrp3 transcription. (**A**) *Prdx6*^−/−^ mLECs showed increased Nlrp3 promoter activity. *Prdx6*^+/+^ and *Prdx6*^−/−^ mLECs were transiently transfected with pGL4-mNlrp3 (−1866/+166); 48 h later, luciferase (LUC) activity was measured. All histograms are presented as mean ± S.D. values derived from three independent experiments. *Prdx6*^+/+^ vs. *Prdx6*^−/−^; * *p* < 0.001. (**B**) Significantly enhanced Nlrp3 transcription was observed in mLECs under oxidative stress. mLECs were transiently transfected with pGL4-mNlrp3 (−1866/+166); 24 h later they were subjected to H_2_O_2_ and/or LPS treatment for 24 h, as indicated in figure. LUC activity was measured and shown. All histograms are presented as mean ± S.D. values derived from three independent experiments. Untreated vs. H_2_O_2_ and/or LPS treated mLECs; * *p* < 0.001. (**C**) Klf9 enhanced Nlrp3 transcription. mLECs were transiently transfected with pGL4-mNlrp3 (−1866/+166) along with pGFP-vector (V) or pGFP-Klf9 (K9) plasmids. After 48 h, the relative LUC activity was monitored. Luciferase assay with Klf9-overexpressing construct significantly increased the transcription of Nlrp3. All histograms are presented as mean ± S.D. values derived from three independent experiments. pGFP-vector vs. pGFP-Klf9; * *p* < 0.001. (**D**) Transactivation assay showed reduced Nlrp3 promoter activity in Klf9-depleted mLECs. LV Sh-control and/or LV *Sh*-Klf9-infected mLECs were transfected with pGL4-mNlrp3 (−1866/+166) plasmid construct along with the Renilla vector; 24 h later, cells were washed and treated with H_2_O_2_ or LPS, as indicated in figure. Nlrp3 promoter activity was measured after 24 h of H_2_O_2_ and/or LPS treatment. All histograms are presented as mean ± S.D. values derived from three independent experiments. LV *Sh*-control vs. LV *Sh*-Klf9, and untreated vs. treated with H_2_O_2_ or LPS; # *p* < 0.05, * *p* < 0.001. (**Di**) Western blot analysis showing the *Klf9*-depleted cells. Total lysate was prepared from lentiviral specific to Klf9 *Sh*RNA- or control-infected mLECs. Equal amounts of protein were immunoblotted with Klf9 antibody. Tubulin was used as an internal loading control.

Several nuclear proteins have been found as activators or repressors of Nlrp3 [96,97,98]. The aberrant expression of Klf9 in aging/oxidative stress acts as a major amplifier of the transcriptional response [42,44,45,50,99,100]. Nonetheless, NF-κB has been shown to be a major player in the induction of the inflammatory pathway by transregulating many components of the Nlrp3 inflammasome activation pathway, including Nlrp3 transactivation [101,102,103]. Because we found that LECs overexpressing Klf9 show increased levels of Nlrp3 mRNA expression (Figure 7), we postulated that Klf9 can act as a transregulator of Nlrp3 transcription. To elucidate this, we assayed Nlrp3 transcriptional activity in *Prdx6*-deficient mLECs (Figure 8A) or mLECs facing oxidative stress (Figure 8B) by transiently transfecting the Nlrp3 gene promoter fused to the reporter gene luciferase (LUC) (−1866/+166 nts). We observed a significant increase in Nlrp3′s transcription in *Prdx6*-deficient mLECs, as well as in mLECs exposed to variable amounts of H_2_O_2_. To confirm the finding of whether Klf9 indeed activates Nlrp3 gene transcription, we utilized LECs over and under-expressing Klf9 and transiently transfected with the Nlrp3 gene promoter fused to LUC and measured the contribution of Klf9 in the induction of Nlrp3 transcription. The experiments using the gain or loss of Klf9 expression showed that Nlrp3 can be transcriptionally regulated by Klf9 (Figure 8C,D). These results, for the first time, show that Nlrp3 could be a new target gene for Klf9-mediated transcription.

### 2.9. Physiological Expression of Klf9 Determined the Fate of mLECs during Oxidative Stress by Controlling Antioxidant Gene Prdx6 Expression

**Figure 9:**
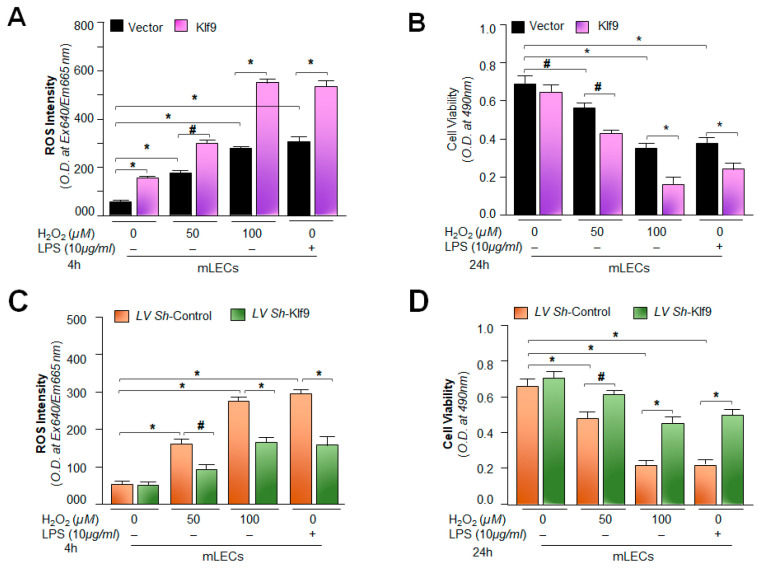
Expression level of Klf9 determined the fate of mLECs during oxidative stress. (**A**,**B**) mLECs overexpressing Klf9 showed the increased accumulation of ROS and reduced viability under oxidative stress. mLECs were overexpressed with pGFP-Vector (Vector) or pGFP-Klf9 (Klf9) plasmid. After 24 h, the transfectants were exposed to different concentrations of H_2_O_2_ or LPS. After 4 h of H_2_O_2_ or LPS exposure, ROS levels (**A**) or at 24 h cell viability (**B**) was determined. Vector vs. Klf9 transfected cells and untreated vs. H_2_O_2_ and/or LPS treated mLECs; # *p* < 0.05, * *p* < 0.001. (**C**,**D**) Depletion of Klf9 suppressed ROS levels and increased cell viability. *Klf9*-depleted mLECs displayed reduced ROS and increased resistance against oxidative stress. An LV *Sh*-Klf9 plasmid was used to deplete the expression of Klf9. After 24 h the infectants were exposed to different concentrations of H_2_O_2_ or LPS. At 4 h of H_2_O_2_ or LPS exposure the ROS level was examined (**C**), or at 24 h cell viability (**D**) was examined. LV *Sh*-control vs. LV *Sh*-Klf9-infected mLECs and untreated vs. H_2_O_2_ or LPS-treated mLECs; # *p* < 0.05, * *p* < 0.001.

To investigate the biological effect of Klf9′s over- or under-expression on mLECs in response to oxidative stress induced by H_2_O_2_, we assessed the levels of ROS prevalence, as well as the viability of mLECs over-expressing Klf9 facing oxidative stress. As expected, mLECs overexpressing Klf9 showed a dramatic increase in ROS levels (Figure 9A), and these mLECs were highly susceptible to H_2_O_2_-induced cell death, as indicated in Figure 9B. Furthermore, we examined whether *Klf9* knock-down in mLECs engenders resistance against H_2_O_2_-induced cell death. To determine this, we depleted Klf9 in mLECs by infecting them with lentiviral (LV) *Sh*-control or LV *Sh*-Klf9, as described in the Materials and Methods. ROS measurement using CellROX deep red reagent and cell viability through MTS assay revealed that *Klf9*-deficiency in mLECs provided resistance against oxidative stress. Altogether, our gain and loss of Klf9 experiments indicated that Klf9 plays an important role in controlling ROS by altering antioxidant gene expression such as Prdx6 (Figure 7). These studies prompted us to conduct experiments to discover if the cellular prevalence of Prdx6 inhibits the process of Nlrp3 inflammasome-mediated inflammatory signaling.

### 2.10. Prdx6 Expression Inhibited Nlrp3 Inflammasome and Its Inflammatory Components Expression/Activation via Alleviating ROS

**Figure 10:**
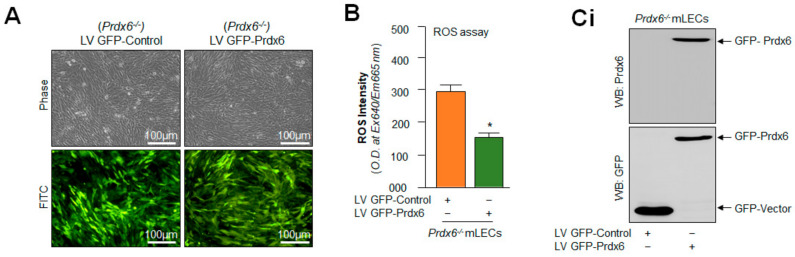

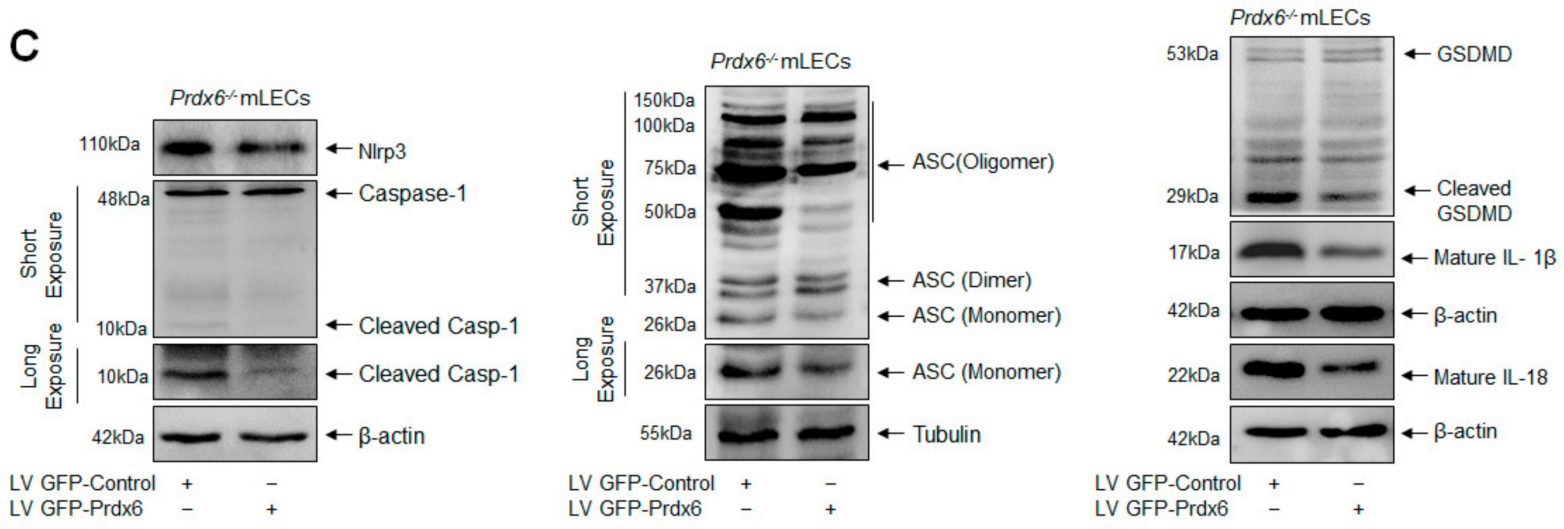
Prdx6 cellular abundance normalized the elevated ROS and Nlrp3 inflammasome gene expression in *Prdx6*^−/−^ mLECs. (**A**) Photomicrograph representing stably infected *Prdx6*^−/−^ mLECs with control (lentiviral (LV) GFP-Control) or GFP (green fluorescent protein)-tagged Prdx6 activation-linked lentiviral (LV GFP-Prdx6). (**B**) ROS intensities were measured using CellROX Red reagent dye in *Prdx6*^−/−^ mLECs infected with LV GFP-Control or LV GFP-Prdx6. Data represent the mean ± S.D. from three independent experiments. LV GFP-control vs. LV GFP-Prdx6 mLECs (*Prdx6*^−/−^), * *p* < 0.001. (**C**) Prdx6 overexpression normalized the increased expression of the Nlrp3 inflammasome, ACS, Casp-1, pyroptosis executer GSDMD, and inflammatory cytokines IL-1β and IL-18 in *Prdx6*^−/−^ mLECs (a model for aging). Total protein was isolated from LV-Control and/or LV GFP-Prdx6-infected *Prdx6*^−/−^ mLECs (**Ci**) and subjected to Western blot, as indicated in figure. Tubulin and/or β-actin was used as the loading control. Data represent the mean ± S.D. from three independent experiments. *Prdx6*^+/+^ vs. *Prdx6*^−/−^ mLECs, * *p* < 0.001.

The results illustrated in Figure 7, Figure 8 and Figure 9 indicated that Prdx6 ablation is a cause of the aberrant Klf9 expression-dependent sustained ROS amplification and the Klf9 activation of aberrant expression of Nlrp3 via its increased transcription, demonstrating that Prdx6 is required to abate the Nlrp3 inflammasome activation pathway. Therefore, we performed experiments to address whether Prdx6 delivery in *Prdx6*-deficient cells subsides the Nlrp3 inflammasome activation-mediated inflammatory pathway. To examine this, *Prdx6*^−/−^ mLECs were expressed with Prdx6 by stably infecting the *Prdx6*^−/−^ mLECs using a lentiviral (LV) GFP-control or LV GFP-Prdx6, as described in the Material and Methods. The LV GFP-control and/or LV GFP-Prdx6-infected *Prdx6*^−/−^ mLECs did not show any phenotypic changes, as indicated in Figure 10A. As expected, *Prdx6*-deficient cells expressing Prdx6 revealed a significant reduction in ROS accumulation (Figure 10B), as evidenced by CellROX deep red reagent assay. In a parallel set of experiments, cultured mLECs were harvested and total proteins were isolated from *Prdx6*^−/−^ mLECs stably infected with LV GFP-control or LV GFP-Prdx6; an equal amount of protein was immunoblotted and analyzed for the Nlrp3 inflammasome related genes, as shown in Figure 10C. Results demonstrated that Prdx6 expression in *Prdx6*-deficient mLECs could abate the aberrant expression and activation of the Nlrp3 inflammasome and its inflammatory components by limiting the ROS levels. The findings demonstrate that Prdx6′s physiological expression is unequivocally critical for protecting cells against oxidative stressors causing Nlrp3 inflammasome activation-mediated inflammatory cell death (pyroptosis), also indicating that its cellular presence is required for maintaining cell health.

## 3. Discussion

Accumulating evidence suggests that the onset of age-related pathogenesis is related to oxidative stress-induced chronic inflammation with advancing age [74]. Indeed, a low-grade inflammatory response has been observed in many elderly persons and has been shown to be connected to the onset of diseases, such as obesity, diabetes, neurodegenerative disease, cardiovascular diseases, cancer, atherosclerosis, and ocular disorders [69,74,104]. Previous discoveries have indicated that elevated ROS-induced oxidative stress is a major culprit for Nlrp3 inflammasome activation-mediated age-related diseases [31,32,89,90,105,106]. In addition, recent published reports support the view that oxidative stress/aging have role in Nlrp3 inflammasome activation-driven inflammatory forms of cell death, like pyroptosis [98,105,106,107]. It is noteworthy to mention that in general, one of the most common characteristics of age-related diseases is a functional decline in the cellular antioxidant response with advancing age. Many studies, including those of our own group, have shown the decline in antioxidant gene expression and activity during aging, resulting in ROS amplification and cell death [4,5,35,65,96,97,108,109]. In this study, we systematically and thoroughly investigated the role of Prdx6 and oxidative stress-driven inflammasome activation and its bioactive components involved in the inflammatory response, and its consequences, by using multiple types of lens epithelial cells. Our data demonstrated that the deficiency of Prdx6 is a cause for ROS accumulation, resulting in an inflammatory form of cell death (pyroptosis). This injurious process includes the aberrant activation of the Nlrp3 inflammasome assembly, i.e., Nlrp3, ASC, Casp-1, IL-1β, IL-18 and GSDMD (Figure 1, Figure 2 and Figure 3). In addition, data revealed that an extrinsic oxidative stimulus further amplified the levels of Nlrp3 inflammasome expression and activation, including its components in *Prdx6*-deficient mLECs. We found that stress induced by H_2_O_2_ led to the increased activation of the Nlrp3 inflammasome and its proinflammatory components, with a significant reduction in Prdx6 in Prdx6-expressing LECs (Figure 4), leading us to argue that Prdx6 is requisite for the physiological regulation of the Nlrp3 inflammasome pathway. It was intriguing to observe that LECs expressing Prdx6 did not display the aberrant activation of the Nlrp3-mediated inflammatory response, supporting our hypothesis that Prdx6 is essential for maintaining cellular homeostasis against ROS-induced Nlrp3 inflammasome activation-mediated injuries. Our results are inconsistent with the findings of recent studies showing that ROS trigger the Nlrp3 inflammasome activation-mediated inflammatory form of cell death (pyroptosis) [9,11,32,90,91,110,111,112], strongly suggesting that Prdx6 acts as a critical quality controller and regulator of the Nlrp3 inflammasome pathway.

Moreover, the loss of antioxidant response has been reported to be a primary cause for aging-related blinding diseases (ARBD) [108,110], including age-related cataract (ARC) [4,5,96,108,110]. Using Prdx6-expressing or *Prdx6*-deficient lenses/LECs, the works of our group and other investigators have shown that *Prdx6*-deficiency leads to oxidative-stress-induced pathological signaling and cell death in the development of ARBD [35,36,42,108,113,114,115,116]. Nevertheless, in many settings, Nlrp3 inflammasome signaling is activated by ROS levels produced in response to intrinsic and extrinsic oxidative stimuli [17]. Figure 1, Figure 2, Figure 3, Figure 4 and Figure 5 show that the oxidative load is directly involved in the improper activation and expression of the Nlrp3 inflammatory response, wherein Prdx6 plays a critical role in controlling the hyperactivated inflammatory response by regulating cellular ROS. The increased expression of Nlrp3, ASC, Casp-1, IL-1β and IL-18 was found in cisplatin-injected kidney tissue. Activated inflammasome complex proteins, Nlrp3, ASC, Casp-1, and a cleaved form of IL-1β and IL-18 have been detected C57BL/6 mice with renal injury after ureteral obstruction, while Nlrp3^−/−^ mice displayed significant less tubular injury in comparison to wild-type mice [117,118], revealing that increased Nlrp3 inflammasome activation and expression are highly damaging to cells/tissues.

The most important question pertaining to this study was to uncover how intracellular ROS could persistently be amplified in aging or during oxidative stress and to identify the factor(s) involved in this process, resulting in the activation of the Nlrp3 inflammasome assembly and inflammatory response. In this context, we recollected our and other investigators’ previously published works that have shown that the aberrant expression of Klf9 in response to aging and oxidative stress causes the Klf9-dependent repression of major antioxidant genes, resulting in the sustained accumulation of ROS. We think that this could be a major event for the existence of a vicious feed-forward regulatory process triggering inflammatory forms of cellular injuries [4,5,42,43,44,45,48,50,108,110,119,120,121]. Indeed, our data demonstrate a significant increase in Klf9 with reduced expression of the major antioxidant genes Prdx6 and Txnrd2 in aging human/mouse LECs (Figure 6). Our results are in agreement with previously published work showing aberrant Klf9 expression-dependent ROS amplification, including the suppression of major antioxidant genes [42,43,44,48,122,123]. Since Klf9 has been known to play role in the activation of proinflammatory signaling [48,51,99,124], we intended to examine if the extrinsic expression of Klf9 enhances the expression of Nlrp3 in LECs. Indeed, it was intriguing to observe that LECs over-expressing Klf9 showed the increased expression of Nlrp3 at the protein as well as mRNA level (Figure 7). Klf9′s increased expression has been reported to activate inflammasome signaling pathways [51,122,123]. Several studies have shown that Klf9 is regulator of redox signaling. Its abundant cellular expression generates abnormal redox signaling, leading to cell death [42,43,44,45]. Very recently, the role of Klf9 has been demonstrated in the regulation of endoplasmic reticulum (ER) stress through facilitating calcium homeostasis. The increased expression of Klf9 transcription by XBP1 has been found under increased ER stress [44]. We have also shown that *Prdx6*-deficiency leads to ER stress, with the increased accumulation of ROS [35,36]. Nevertheless, recent evidence reveals the involvement of ER stress in the induction of the Nlrp3 inflammasome activation pathway [125]. An aberrant increase in ER stress can stimulate increased ROS production and failure of calcium homeostasis, leading to cell damage. It has now been shown that ER-stress-driven Nlrp3 inflammasome activation is linked to the etiopathologies of various inflammatory disorders [126]. Further, mitochondria are highly active organelles and release maximum amounts of intracellular ROS [62]. Mitochondrial disintegration and dysfunction acts as an upstream activator of Nlrp3 via ROS generation, and also acts as a platform for inflammatory signaling [127,128,129]. Moreover, our results revealed the increased ROS generation and aberrant Nlrp3 expression with reduction in antioxidant gene Prdx6 in SRA-hLECs, as well as in mLECs overexpressing Klf9 (Figure 7). These data revealed that Klf9′s increased expression in LECs during aging/oxidative stress is a major cause for dysregulation of redox signaling, resulting in the inflammatory response-mediated onset of pathobiology.

In this study, we found that modulation in the Klf9-mediated expression of Nlrp3 in LECs was at the transcript level. This indicates the plausible involvement of Klf9 in regulating Nlrp3 gene transcription. This nuclear protein has been reported to be a critical element involved in the modulation of a redox system [44,50,51,130]. Our group and other investigators have reported that Klf9 acts as a pro-oxidant factor, and can be aberrantly regulated by Nrf2′s excessive accumulation in the nucleus during oxidative stress [42,43,45]. Mechanistically, our transactivation experiments demonstrated that indeed, Klf9 altered redox signaling-mediated Nlrp3 inflammatory signaling at transcriptional levels, and it does so by upregulating Nlrp3 transcription (Figure 8). In addition, we found that the increased oxidative stress-dependent upregulation of Nlrp3 was related to the increased abundance of Klf9 in LECs. Thus, we showed for the first time that Nlrp3 can be a new target gene for Klf9-mediated regulation. Our study showed that, indeed, Klf9 expression was upregulated during oxidative stress, leading to increase levels of Nlrp3, one of the very important findings of the present study. We also identified that LECs overexpressed with Klf9 displayed increased Nlrp3 expression and these cells were highly vulnerable to H_2_O_2_- and LPS-induced cell damage (Figure 9).

Moreover, previously, we reported that SRA-hLECs overexpressing Klf9 leads to a decline in antioxidant gene expression, and these SRA-hLECs were prone to H_2_O_2_ and/or higher concentrations of sulforaphane (SFN)-induced oxidative damage [42,43]. Furthermore, our data from the loss-and-gain Klf9 experiment showed modulation in Nlrp3 promoter activity (Figure 9), suggesting that a cellular abundance of Klf9 regulates Nlrp3 inflammasome-mediated inflammatory signaling depending upon the cellular status and microenvironment. Furthermore, *Klf9*-deficiency in mLECs protects the LECs against oxidative-stress-induced cell damage by normalizing ROS production (Figure 9). It has been reported that *Klf9*-knockdown protects cardiomyocytes against ischemic injury by reducing ROS with the upregulation of Txnrd2 [48,51,124]. Klf9 over-expression increased cell death; conversely, *Klf9*-knockdown helps resist ischemic injury and protect the cardiomyocytes [131]. Further, inflammation and oxidative stress are the main features of diabetic cardiomyopathy (DCM), and Klf9 expression was found to be upregulated in DCM. Klf9 over expression aggravates inflammation, oxidative stress, and cardiac dysfunction in DCM, while *Klf9*-knockdown ameliorates the process. We observed upregulated Nlrp3 transcription in *Prdx6*^−/−^ mLECs, a model for aging, as well as mLECs facing oxidative stress (Figure 8). It is notable that the delivery of Prdx6 or the enhanced expression of Prdx6 using inductive therapy, or known FDA approved drugs such as Metformin and Hydralazine protect the cells against oxidative stress by normalizing ROS generation [4,5,42,43,45,65]. In this work, we observed that the overexpression of Prdx6 abated the elevated activation of the Nlrp3 inflammasome activation pathway and subsided the activation of inflammatory molecules in *Prdx6*^−/−^ mLECs (Figure 10). Several studies have shown that antioxidants like acetaminophen or N-acetylcysteine (NAC), including dietary supplements, can blunt the LPS or oxidative-stimulated Nlrp3 inflammasome activation in vitro and in vivo [96,132,133,134,135]. In addition, it has been found that rosnarinic acid has antioxidant and anti-inflammatory effects, by which it protects from acute kidney injury caused by the well-known anti-cancer drug Cisplatin [117]. Moreover, existing reports as well as the results of this study have established that Prdx6 is a regulator of the Nlrp3 activation-mediated inflammatory response, and Prdx6 expression is essential to block the inflammatory process.

## 4. Materials and Methods

### 4.1. Cell Culture

#### 4.1.1. Mouse Lens Epithelial Cells (mLECs) Isolated from Lenses of C57BL/6 Mice

Three types of mLECs were used: (1) a cell line of wild-type (*Prdx6*^+/+^) mLECs (2) *Prdx6*-targeted mutant (*Prdx6*^−/−^) mLECs, and (3) primary mLECs isolated from a C57BL/6 mouse.

All animal experiments followed the recommendations set forth in the “Statement for the Use of Animals in Ophthalmic and Visual Research” by the Association for Research in Vision and Ophthalmology. All studies on animals were approved by the Institutional Animal Care and Use Committee (IACUC), University of Nebraska Medical Center (UNMC), Omaha, NE. All animals were maintained under specific pathogen-free conditions in an animal facility. LECs isolated from *Prdx6*-targeted mutants (*Prdx6*^−/−^) and wild-type (*Prdx6*^+/+^) mice were generated and maintained in Dulbecco’s modified Eagle’s medium (DMEM; Invitrogen, Carlsbad, CA, USA) with 10% FBS (Atlanta Biologicals, Inc., Flowery Branch, GA, USA) as described earlier [35]. We used *Prdx6*^−/−^ mutant mice, from the same fully inbred C57BL/6 background, and wild-type mice of the same sex and age (*Prdx6*^+/+^). This minimizes the variation due to genetic background. All animals were maintained under specific pathogen-free conditions in an animal facility. LECs were isolated from mice of identical age, and Western blot analysis was carried out to confirm the presence of αA-crystalline [35,136], a specific marker of LECs. Cells from 3–5 passages were used for the experiments. Cells were harvested in a 96-well plate or 100 mm culture dishes for 12–14; these cells were washed with 1× PBS and exposed to different concentrations of H_2_O_2_ and/or LPS in DMEM media containing 0.2% BSA, as indicated in the figures and legends.

Isolation of primary mouse lens epithelial cells: Different ages of C57BL/6 male or female mice were obtained from Charles River laboratories, MA, USA, and were maintained at a stable temperature (22 ± 2 °C) and humidity (55 ± 5%). The mice were sacrificed using cervical dislocation and lens were collected for isolation of lens epithelial cells. Briefly, the capsule was trimmed before explanting in 35 mm culture dishes precoated with collagen IV containing a minimum amount of DMEM, supplemented with 15–20% fetal bovine serum (FBS) with a brief modification [35,137,138]. Capsules were spread by forceps with cell layers upward on the surface of plastic Petri dishes. Culture explants were trypsinized and re-cultured. Cell cultures attaining 90 to 100 percent confluency were trypsinized and used for this study [35,42,43]. Western blot analysis was carried out to validate the presence of αA-crystallin, a specific marker for LEC identity. ROS, Western blot and mRNA analyses were carried out with mLECs directly separated from lens to avoid the cell culture effect.

#### 4.1.2. SRA-Human Lens Epithelial Cells (hLECs) and Primary hLECs Culture and Maintenance

Two types of hLECs were used: (1) a hLEC cell line (SRA01/04) immortalized with SV40 [136], and (2) primary human LECs isolated from deceased persons of variable ages. To avoid confusion, the remaining text will designate the immortalized LECs as SRA-hLECs and the primary human LECs of different ages as hLECs or primary hLECs.

The SRA-hLECs were derived from 12 infants who underwent surgery for retinopathy of prematurity [136] (a kind gift of the late Dr. Venkat N. Reddy, Eye Research Institute, Oakland University, Rochester, MI, USA). These cells were maintained in Dulbecco’s Modified Eagle Medium (DMEM, Invitrogen, Waltham, MA, USA) with 15% fetal bovine serum (FBS, Atlanta Biologicals, Atlanta, GA, USA), 100µg/mL streptomycin, and 100 µg/mL penicillin in a 5% CO_2_ environment at 37 °C, as described previously [65]. SRA-hLECs were harvested in 100 mm culture dishes overnight; these cells were washed with 1× PBS and exposed to different concentration of H_2_O_2_ and/or LPS in DMEM media containing 0.2% BSA, as indicated in the figures and legends. Gene expressions were examined using Western blot and RT-qPCR analysis, as shown in the figures and legends.

Primary hLECs were isolated from normal eye lenses of deceased persons or healthy donors of different ages (16 years (y) and 65 y), obtained from the Lions Eye Bank, Nebraska Medical Center, Omaha, NE, USA. These primary hLECs were used for experimentation in the present study. According to regulation HHS45CFR 46.102(f), studies involving material from deceased individuals are not considered human-subject research as defined under 45CFR46.102(f) 10(2) and do not require IRB oversight. Briefly, the capsule was trimmed before explanting in 35 mm culture dishes precoated with collagen IV, containing a minimum amount of DMEM containing 15–20% fetal bovine serum (FBS), with a brief modification [35,137,138]. Capsules were spread by forceps with cell layers upward on the surface. Culture explants were trypsinized and cells were cultured. Cell cultures showing 90 to 100 percent confluency were trypsinized and used for experiments. Western analysis was used to validate the presence of αA-crystallin, a specific marker for LEC identity.

### 4.2. Quantitation of Intracellular Reactive Oxygen Species (ROS)

#### 4.2.1. ROS Level by H2-DCF-DA in LECs

The intracellular ROS level was measured by use of fluorescent dye dichlorofluorescin diacetate (H2-DCF-DA), a nonpolar compound that is converted into a polar derivative (dichlorofluorescein) by cellular esterase after incorporation into cells [4,5,42]. *Prdx6*^+/+^ and *Prdx6*^−/−^ mLECs were trypsinized and seeded in 96-well plates overnight. These cells were washed with 1× PBS and then exposed with H_2_O_2_ in DMEM media containing 0.2% BSA, as indicated in the figures and legends. After 4 h of H_2_O_2_ exposure, the medium was replaced with Hank’s solution containing 10 µM H_2_-DCF-DA dye, and cells were incubated for 30 min. A Spectra Max Gemini EM (Mol. Devices, Sunnyvale, CA, USA) detected intracellular fluorescence with excitation (Ex) at 485 nm and emission (Em) at 530 nm.

C57BL/6 mice of different ages (2 months (M), 8 M and 19 M) were obtained from Charles River laboratories, MA, USA and maintained as noted above. The mice were sacrificed using cervical dislocation and lenses were collected and immediately frozen at −80 °C. Aging hLECs were directly separated from lens and stored at −80 °C. ROS levels were measured according to our published protocol [4,5,42]. Briefly, mouse lenses/hLECs were thawed on ice and homogenized (100 mg/mL) in freshly prepared homogenization buffer [50 mM Phosphate buffer containing 1 mM Ethylenediaminetetraacetic acid (EDTA), 0.5 mM phenylmethylsulfonyl fluoride (PMSF), 1 µM Pepstatin, 80 mg/L Trypsin Inhibitor, pH 7.4]. H2-DCF-DA dye was added to freshly prepared lens homogenate in a 96-well plate to achieve a 30 µM final concentration. After 30 min of incubation at 37 °C, intracellular fluorescence was detected with excitation (Ex) at 485 nm and emission (Em) at 530 nm by a Spectra Max Gemini EM (Mol. Devices, Sunnyvale, CA, USA).

#### 4.2.2. Quantitation of Intracellular ROS Level by CellROX^®^ Deep Red Reagent

ROS levels were measured according to the company’s protocol (CellROX^®^ deep red oxidative stress reagent, Catalog No. C10422) [42,43]. In brief, pGFP-vector and pGFP-Klf9-transfected mLECs and/or an LV *Sh*-Control and LV *Sh*-Klf9 infected mLECs (10 × 10^3^) were cultured in 96-well plate; 24 h later cells were exposed to different concentration of H_2_O_2_. After 4 h, CellROX deep red reagent was added, with a final concentration of 5 µM, and cells were incubated at 37 °C for 30 min. Media containing CellROX deep red reagent were removed and fixed with 3.7% formaldehyde. After 15 min, the fluorescence signal was measured at Ex640 nm/Em665 nm with a Spectra Max Gemini EM (Molecular Devices, Sunnyvale, CA, USA).

### 4.3. Protein Isolation and Western Blotting

Total cell lysates of LECs were prepared in ice-cold radioimmune precipitation buffer (RIPA buffer), and immunoblot analysis was performed as described previously [4,5,34,35,42]. The membranes were probed with anti-Prdx6 antibody (LF-PA0011, Ab Frontier, Seoul, Republic of Korea), Nlrp3 (#PA5−79740, ThermoFisher Scientific, Waltham, MA, USA), Caspase-1 (#24232S and #3866S, Cell Signaling Technology, Danvers, MA, USA), ASC (#13833S and #67824S, Cell Signaling Technology, Danvers, MA, USA), IL-1β (#122452, Cell Signaling Technology, Danvers, MA, USA), IL-18 (#57058S and # 54943S, Cell Signaling Technology, Danvers, MA, USA), Gasdermin D (#39754S, Cell Signaling Technology, Danvers, MA, USA), Klf9 (ab177158), IL-1β (sc-52012, Santa Cruz Biotechnology, Dallas, TX, USA), ASC (sc-271054, Santa Cruz Biotechnology, Dallas, TX, USA) and β-actin (A2066, Sigma-Aldrich, St. Loius, MO, USA)/Tubulin (ab44928, Abcam, Boston, MA, USA) as an internal control, to monitor those protein expressions. After the secondary antibody (sc-2354 and sc-2768, Santa Cruz Biotechnology, Dallas, TX, USA), protein bands were visualized by incubating the membrane with luminol reagent (sc-2048; Santa Cruz Biotechnology, Dallas, TX, USA) and images were recorded with a FUJIFILM-LAS-4000 luminescent image analyzer (FUJIFILM Medical Systems Inc., Hanover Park, IL, USA).

### 4.4. RNA Isolation and mRNA Analysis of Mouse or Human LECs Using RT-qPCR

Total RNA was isolated from LECs using the single-step guanidine thiocyanate/phenol/chloroform extraction method (Trizol Reagent, Invitrogen). A weight of 0.5 to 5 micrograms of total RNA was transcribed to cDNA using Superscript II RNAase H-reverse-transcriptase to examine the levels of Prdx6, Klf9, Trxnd2, Nlrp3, ASC, Caspase-1, IL-1β, IL-18, GSDMD and β-actin, as mentioned in Table 1. Real-time quantitative PCR (RT-qPCR) was conducted with SYBR Green Master Mix (Roche Diagnostic Corporation, Indianapolis, IN, USA) in a Roche^®^ LC480 Sequence detector system (Roche Diagnostic Corporation) under PCR conditions of 5–10 min (min) hot start at 95 °C, followed by 45–55 cycles of 10 s (sec) at 95 °C, 30 s at 60 °C and 10 s at 72 °C. Sequences of the primers used in the study are shown below in Table 1.

The relative quantity of the mRNA expression was obtained using the comparative threshold cycle (CT) method. The expression levels of target genes were normalized to the levels of β-actin as an endogenous control in each group.

### 4.5. Mouse and Human Caspase-1 ELISA Assay

Caspase-1 activity was measured in LECs using a Caspase-1 (Mouse) ELISA Kit (Catalog #E4180-100; BioVision, Milpitas, CA, USA) and Caspase-1 (Human) ELISA Kit (Catalog #E4588-100; BioVision, Milpitas, CA, USA) following the company’s protocol. For the standard, lyophilized Caspase-1 was reconstituted in standard/sample dilution buffer to make 2000 pg/mL or 5000 pg/mL of standard stock solution in the mouse and human kit, respectively. To prepare the standard curve within the range, 2-fold serial dilutions of the top standards were used to perform the assay. For *Prdx6*^+/+^ and *Prdx6*^−/−^ mLECs samples, cells were cultured in serum-free media for 48 h. Cell-culture media were removed, and cells were collected after washing with 1× PBS twice, and the cellular extract was prepared using RIPA buffer. For the primary LEC sample: LECs were directly separated from lenses and washed with 1× PBS. Cellular extracts were prepared using RIPA buffer and equal amounts of protein lysate were used to detect the status of Caspase-1. All reagents and standards/samples were brought to room temperature 30 min prior to the assay. Plate was washed with a 1× wash solution, and the appropriate amount of 100 µL of the standards/samples were added to the well and covered following 90 min incubation at 37 °C. Standards/samples were removed, and 100 µL of Biotin detection was added, followed by 60 min incubation at 37 °C. After 3 times of washing with 1× wash solution, 100 µL of SABC working solution was added to each well and incubated for 30 min at 37 °C. After 5 times of washing with 1× wash solution, 90 µL of the TMB substrate was added to each well and incubated for 15–30 min at 37 °C away from light. A volume of 50 µL of stop solution was added once, turning the solution blue, and its optical density (O.D) was measured at 450 nm with a Spectra Max Gemini EM (Molecular Devices, Sunnyvale, CA, USA).

### 4.6. Mouse and Human IL-1β ELISA Assay

IL-1β levels were measured using Human IL-1β ELISA Kit (Catalog #ab214025, Abcam) and Mouse IL-1β ELISA Kit (Catalog #ab197742, Abcam) following the manufacturer’s instructions. For the standard, lyophilized IL-1β was reconstituted in 500 µL Sample Diluent NS and/or 1× cell Extraction Buffer PTR to make the 200 pg/mL or 4000 pg/mL of standard stock solution in the mouse and human sample, respectively. Eight serial dilutions were prepared from the stock standard to perform the assay. The following steps were taken for sample preparation: (1) The collected cell culture supernatant at 48 h was centrifuged for 20 min, and then the clear supernatant was transferred to fresh tubes and stored at −80 °C. (2) Growth media were removed, and cells were washed twice with 1× chilled PBS and collected using a scraper and centrifuged at 500× *g* for 5 min at 4 °C, and excess PBS was removed. Cells were resuspended in chilled 1× Cell Extraction Buffer PTR and incubated on ice for 20 min, following high centrifugation for 20 min at 4 °C. Supernatants transferred into fresh tubes and aliquots were immediately stored at −80 °C. For assay, all solutions were brought to room temperature. A volume of 50 µL of standard/samples were loaded to the wells, and then 50 µL of antibody cocktail was added to the well following the incubation at room temperature for 1 h on a plate shaker. After three times washing with 1× wash buffer PT, 100 µL of TMB Development Solution was added to each well and incubated for 10–15 min in dark on a plate shaker set to 400 rpm. A volume of 100 µL of stop solution was added to each well and mixed for 1 min on shaker plate. Endpoint O.D was measured at 450 nm with Spectra Max Gemini EM (Molecular Devices, Sunnyvale, CA, USA).

### 4.7. Mouse and Human IL-18 ELISA Assay

IL-18 levels were measured as mentioned in the manufacturer’s protocol (Human IL-18 ELISA Kit, Catalog #ab215539, Abcam and mouse IL-18 ELISA Kit, Catalog #ab216165, Abcam). For standard preparation, lyophilized IL-18 was reconstituted in 500 µL Sample Diluent NS and/or 1× cell Extraction Buffer PTR to make the 5465 pg/mL or 8000 pg/mL of standard stock solution in the mouse and human samples, respectively. Serial dilutions were prepared from stock standard for making the standard curve. For sample preparation, the following steps were taken: (1) Cells were cultured, supernatant was collected at 48 h, and clear supernatant was transferred to fresh tubes after centrifugation for 20 min and immediately stored at −80 °C. (2) Cells were washed twice with 1× chilled PBS after removing the cell culture medium. Cells were collected and resuspended in chilled 1× Cell Extraction Buffer and incubated on ice for 15 min. Cell suspension was highly centrifuged and clear lysate was transferred to the fresh tube. Assay samples were immediately stored at −80 °C. All solutions were brought to room temperature prior to the assay. Standard/samples (50 µL) was added to the wells, and then antibody cocktail (50 µL) was added to the well. Plate wells were covered and incubated on a plate shaker set at 400 rpm. Wells were washed three times with 1× wash buffer PT. Excess liquid was removed prior to the addition of TMB Development Solution (100 µL) and plate wells were incubated for 10–15 min in the dark on a plate shaker set to 400 rpm. Plate wells mixed on a shaker plate after the addition of stop solution (100 µL) and O.D were measured at 450 nm with Spectra Max Gemini EM (Molecular Devices, Sunnyvale, CA, USA).

### 4.8. Plasmids or Constructs and Lentiviral (LV) Infection

pGFP-Vector (Clontech, San Jose, CA, USA) and pGFP-Klf9 (OriGene, Rockville, MD, USA) were purchased. CopGFP control lentiviral particle (LV *Sh*-Control, sc-108084, Santa Cruz Biotechnology, Dallas, TX, USA) and Klf9/GFP ShRNA (LV *Sh*-Klf9, sc-37716-VS, Santa Cruz Biotechnology, Dallas, TX, USA) were purchased from Santa Cruz Biotechnology, and mLECs were infected following the company’s protocol and as published earlier [42,43]. In brief, mLECs were cultured in a 6-well plate in complete medium. After 24 h, the media were replaced with 2 mL of polybrene (sc-134220, Santa Cruz Biotechnology, Dallas, TX, USA) at a final concentration of 5 µg/mL. *Sh*-Control and *Sh*-Klf9 lentiviral particles were added to the culture to infect the cells, mixed by swirling and incubated overnight. After 24 h, polybrene-containing media were removed and fresh complete media were added. Infected mLECs were split and incubated for 24 h, and then treated with puromycin dihydrochloride (sc-108071, Santa Cruz Biotechnology, Dallas, TX, USA) selection marker for stable cell lines. These stable cell lines were used for the current study.

### 4.9. Construction of Nlrp3 Promoter—Luciferase and Promoter Activity

The 5′-flanking region of mouse Nlrp3 gene ranging from −1866 to +166 bps DNA fragment was isolated from mouse genomic DNA by using an Advantage^®^ Genomic LA polymerase Mix (Catalog number 639152, Takara Bio, Inc., San Jose, CA, USA). A DNA fragment of −1866 to +166 bps was constructed by ligating this DNA fragment into a pGL4.21 reporter vector using *SacI* and *XhoI* sites. The plasmid was amplified and sequenced. Primers used for isolating the genomic DNA fragment were as follows: forward primer (*SacI* site): 5′-AAAA*GAGCTC*CTTCAGCCTTAGCATTTG-3′; reverse primer (*XhoI* site) 5′-AAAA*CTCGAG*CTTGATCCAGACGTATGTCC-3′. LECs were transfected with the above-mentioned plasmids along with Renilla, pRL-TK vector (Promega, Madison, WI, USA). Transfectants treated with LPS or H_2_O_2_ were processed for luc-assay. Luciferase activity was measured using a Dual-Glo luc assay system with a 96-well plate (Promega) and normalized with Renilla O.D.

### 4.10. Statistical Analysis

All experiments involved at least three independent biological replicas. Results are expressed as the mean ± standard deviation (S.D.). Sigma plot 12.5 software was used for statistical analyses. Statistical analysis was carried out using Student’s *t*-test and/or one-way ANOVA to assess the significance of outcomes between groups. Statistical significance between the control and treatment groups was assessed and *p* values < 0.05 and <0.001 were considered statistically significant.

## 5. Conclusions

**Figure 11:**
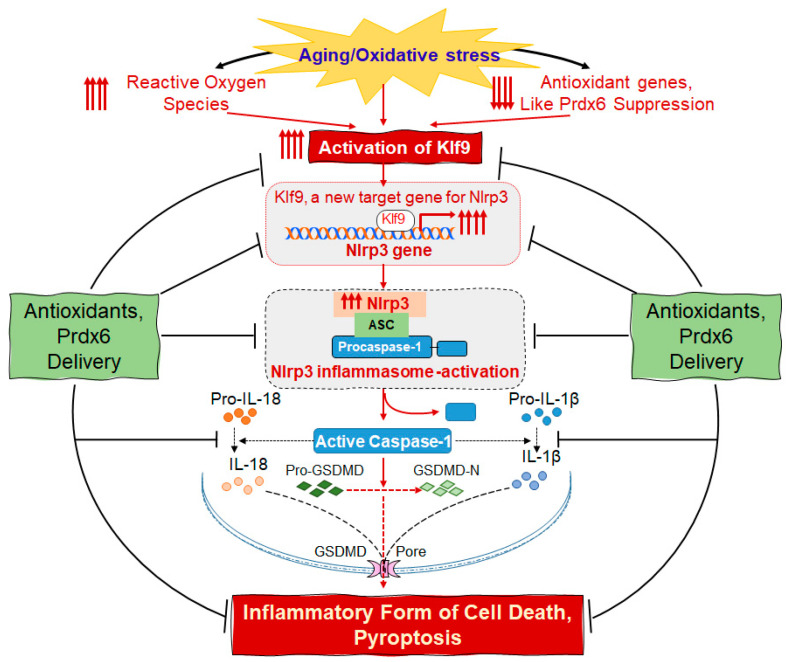
Scheme showing the molecular mechanism of ROS amplification and the ROS-driven Nlrp3 inflammasome activation pathway in *Prdx6*-deficient lens cells. Aberrant activation of Nlrp3 inflammasome in cells or tissues due to increased amplification of ROS contributes to the pathology of age-related diseases, during which levels of antioxidants like Prdx6 play a pivotal role to balance redox homeostasis. Figure 11 elucidates the effects of Prdx6 on sustained ROS accumulation-driven expression and how Prdx6 could regulate the activation of the Nlrp3 inflammasome and its bioactive molecule-mediated inflammatory responses, leading to an inflammatory form of cell death. The principal findings of this study illustrated the presence and mechanism of lens cell-specific *Prdx6*-deficiency in regulating oxidative-induced aberrant Nlrp3 inflammasome activation-mediated death signaling in aging cells. We showed that *Prdx6*-deficiency caused ROS accumulation in aging cells by the Klf9 suppression of Prdx6 [42,43], leading to Klf9-dependent aberrant Nlrp3 expression, a new target gene for Klf9. This feed-forward phenomenon within cellular microenvironment during aging can result in increased Nlrp3/Casp-1/GSDMD-mediated inflammatory signaling and cell damage. Thus, our study suggests that strategies preventing ROS and Klf9 accumulation by means of antioxidants, like Prdx6, could be a promising approach to ameliorate age-related diseases, including cataract formation.

In conclusion, for the first time, we reported that *Prdx6*-deficiency is a cause of the aberrant upregulation and activation of the Nlrp3 inflammasome by Klf9 in lens cells, resulting in the inflammatory form of cell death pyroptosis. Our data showed that *Prdx6*^−/−^ mLECs and aging LECs isolated from different ages of mouse and healthy human subjects had a higher ROS prevalence, had an Nlrp3 inflammasome activation pathway with its bioactive components, and were responsible for an inflammatory form of cell death in aging LECs due to a loss of Prdx6. We found that a loss of Prdx6 in aging LECs led to the oligomerized cluster of Nlrp3-ASC-cleaved Casp-1 assembly, which resulted in Nlrp3 inflammasome activation and its bioactive inflammatory components, GSDMD, IL-1β and IL-18. Mechanistically, our data revealed the existence of the Klf9-dependent feedforward process causing the suppression of Prdx6 and excessive ROS accumulation, resulting in increased Klf9-medaited Nlrp3 expression and activation. Herein, we propose that combination therapies, i.e., inhibitors(s) of Klf9 or Nlrp3, and the delivery of Prdx6 could be a promising therapeutic strategy to prevent/delay the Nlrp3 inflammasome activation-mediated pathobiology causing age-related diseases; also, our work paves the way for future research to delineate the specific role of Prdx6/ROS in connection to the regulation of Nlrp3 inflammasome-activation and disease onset (Figure 11).

## Figures and Tables

**Table 1:** Primer sequence for RT-qPCR.

Gene	Forward Primer (5′ to 3′)	Reverse Primer (5′ to 3′)
mNlrp3	TCACAACTCGCCCAAGGAGGAA	AAGAGACCACGGCAGAAGCTAG
mASC	CTGCTCAGAGTACAGCCAGAAC	CTGTCCTTCAGTCAGCACACTG
mCaspase-1	GGCACATTTCCAGGACTGACTG	GCAAGACGTGTACGAGTGGTTG
mIL-1β	TGGACCTTCCAGGATGAGGACA	GTTCATCTCGGAGCCTGTAGTG
mIL-18	GACAGCCTGTGTTCGAGGATATG	TGTTCTTACAGGAGAGGGTAGAC
mGSDMD	GGTGCTTGACTCTGGAGAACTG	GCTGCTTTGACAGCACCGTTGT
mPrdx6	TTCAATAGACAGTGTTGAGGATCA	CGTGGGTGTTTCACCATTG
mKlf9	CTACAGTGGCTGTGGGAAAGTC	CTCATCCGAGCGCGAGAACTTT
mTxnrd2	GCCATTGGAGATGTTGCTGAGG	CACAGCCATACTCCAGTGGTGT
Mβ-actin	CTAAGGCCAACCGTGAAAAG	ACCAGAGGCATACAGGGACA
hNlrp3	GGACTGAAGCACCTGTTGTGCA	TCCTGAGTCTCCCAAGGCATTC
hASC	AGCTCACCGCTAACGTGCTGC	GCTTGGCTGCCGACTGAGGAG
hCaspase-1	GCTGAGGTTGACATCACAGGCA	TGCTGTCAGAGGTCTTGTGCTC
hIL-1β	CCACAGACCTTCCAGGAGAATG	CCTTGATGTTATCAGGAGGATTCA
hIL-18	GATAGCCAGCCTAGAGGTATGG	GATAGCCAGCCTAGAGGTATGG
hPrdx6	GCATCCGTTTCCACGACT	TGCACACTGGGGTAAAGTCC
hKlf9	CTGGTTGCTGGGACTGTAGC	GTTTTCCAGCTCCCAAACAG
hTxnrd2	GCACCTTTGACACCGTCCTGTG	CACCAGGATCTTCTGAGTGTCG
Hβ-actin	CCAACCGCGAGAAGATGA	CCAGAGGCGTACAGGGATAG

## Data Availability

Data are contained within the article.
